# DiffMethylTools: a toolbox for the detection, annotation, and visualization of differential methylation

**DOI:** 10.26508/lsa.202603765

**Published:** 2026-07-06

**Authors:** Houssemeddine Derbel, Evan Kinnear, Justin J-L Wong, Qian Liu

**Affiliations:** 1 Nevada Institute of Personalized Medicine, University of Nevada , Las Vegas, NV, USA; 2 Epigenetics and RNA Biology Laboratory, School of Medical Sciences, The University of Sydney , Camperdown, Australia; 3 School of Life Sciences, College of Sciences, University of Nevada , Las Vegas, NV, USA

## Abstract

DNA methylation is a fundamental epigenetic mechanism, and its significant changes (i.e., differential methylation) regulate gene expression, cell-type specification, and disease progression without altering the underlying DNA sequence. Differential methylation has traditionally been detected by statistical comparison of two groups using existing tools and has supported a wide range of downstream analyses in human disease studies. However, few toolboxes efficiently integrate robust detection, annotation, and visualization of differential methylation, and the reproducibility of detected signals across tools remains limited. Moreover, existing methods have rarely been evaluated on long-read methylomes, despite their increasing availability. Here, we present DiffMethylTools, an end-to-end framework designed to address analytical and computational challenges in differential methylation studies. DiffMethylTools generates robust differential methylation detection with comprehensive annotation and visualization modules to streamline analysis workflows. Benchmarking across six datasets, including three long-read methylomes, shows that DiffMethylTools outperforms four widely used tools in detecting differential methylation, with improved overall performance and reproducibility. In addition, DiffMethylTools offers compatibility with upstream methylation-calling pipelines, and facilitates downstream biological interpretation through flexible annotation and visualization capabilities.

## Abstract (precis)

An integrated pipeline accurately identifies differentially methylated loci and regions, linking these epigenetic signals to biological functions through robust annotation and visualization.

## Introduction

DNA methylation, such as 5-Methylcytosine (5mC) and 5-hydroxymethylcytosine (5hmC), is a compulsory and fundamental epigenetic process without altering the underlying sequence (1). It widely occurs in prokaryotes and eukaryotes (2) and regulates gene expression and cellular functions (1). For example, in mammal genomes, it plays a crucial role in epigenetic regulation and is essential for biological processes (3) such as genomic imprinting (4), X-chromosome inactivation (5), and suppression of transposable elements (6). DNA methylation also stably regulates cell type and tissue specific biological functions of an identical genome in an individual (7), highlighting the importance of DNA methylation in biological systems.

Whole genome profile of DNA methylation could be detected via different sequencing techniques, such as widely-used bisulfite sequencing (BS-seq) and reduced representation bisulfite sequencing (8), which used bisulfite conversion and short-read sequencing to detect methylated and unmethylated sites. Recently, third-generation sequencing technologies, including Nanoball Sequencing (9) (Illumina’s Nanoball Technology), Helicos Sequencing (10) (Helicos BioSciences), Single-Molecule Real-Time Sequencing (11) from Pacific Biosciences, and Oxford Nanopore Technologies (12, 13), were proposed to improve the methylation detection especially in low-complexity genomic regions and enable the detection of non-5mC DNA modifications.

The advancement of methylation detection stimulated the detection of differential methylation patterns across various biological process and human diseases (4, 14, 15, 16). These differential methylation patterns offered valuable insights into gene expression regulation influenced by environmental factors (17), developmental processes (18), or disease states (19), and facilitating the discovery of biomarkers for early disease diagnosis (20), prognosis (21), and monitoring of disease progression (22) or treatment response (23, 24).

There are two types of differential methylation patterns, differentially methylated loci (DMLs) for differential CpG sites or positions, and differentially methylated regions (DMRs) which are a cluster of DMLs and reflect synergistic effect of DMLs. These methylation patterns could be detected by various computational tools using whole-genome methylation profiles as input. These tools could be classified into four categories according to the techniques used. One category of tools used simple statistical models like Fisher’s exact test and *t* tests to compare methylation frequencies between conditions. These methods, such as DMRfinder (25), and eDMR (26), were fast and worked well with high coverage data. However, their performance is not robust because they do not model biological replication structure or coverage-dependent variance in methylation sequencing data. The second category of computational tools used linear model (GLM)-based methods, and included methylKit (27), methylSig (28), DSS-single (29), DSS (30, 31), DMRcaller (32), MOABS (33), RADMeth (34), DMAP (35), RnBeads (36, 37), and COHCAP (38). These methods modeled methylation counts or methylation levels using binomial or beta-binomial distributions to account for both biological variability and technical noise. These methods were generally well-suited for high-coverage sequencing datasets, and their performance tended to degrade on low-coverage datasets where variance estimates become unreliable. The third category of computational tools, such as such as BSmooth (39) and BiSeq (40), used smoothing process to reduce random noise by aggregating nearby CpG sites and were effective on low coverage data. However, smoothing did not show much improvement compared with other tools (41). The fourth category of computational tools used Hidden Markov Model (42) (HMM) (such as HMM-DM (43), HMM-Fisher (44) and Bisulfighter (45)) to model discretized methylation states and transitions along the genome by exploiting short-range CpG dependence. However, they ignored complex CpG structures, and employed stationary transition dynamics, limiting parameter transferability and generalizability. In summary, diverse assumptions in different methods led to poor overlap of detected differential methylation patterns. Besides, most of these tools either lack built-in modules or offer limited support for annotation and visualization. A few packages, such as DMRcaller, MethylSig, and COHCAP, offered downstream processes of plotting local methylation profile, but they typically provided either basic annotation or rudimentary visualization. That is, most tools remained focused exclusively on differential methylation detection, leaving users to assemble separate, often fragmented pipelines for biological interpretation and visualization. Thus, a toolbox is needed to simplify and speedup the detection, annotation, and visualization of differential methylation patterns.

To address these issues, we designed DiffMethylTools, a single-command-for-all tool for differential analysis. DiffMethylTools is flexible to various input formats of DNA methylation and performs reliable detection of DMLs and DMRs as well as annotation and visualization in a single command. We evaluated DiffMethylTools on three WGBS short-read data and three long-read sequencing data generated in our lab, and compared its performance against four widely used tools, MethylKit, DSS, MethylSig, and bsseq. The results demonstrated that DiffMethylTools overall outperformed the existing tools to detect significant methylation patterns. DiffMethylTools thus offered straightforward investigation of differential methylation patterns and will benefit human disease studies to investigate epigenetic roles in disease development and progression. DiffMethylTools is publicly available via https://github.com/qgenlab/DiffMethylTools.

## Results

### Overview of DiffMethylTools

**Figure 1.**
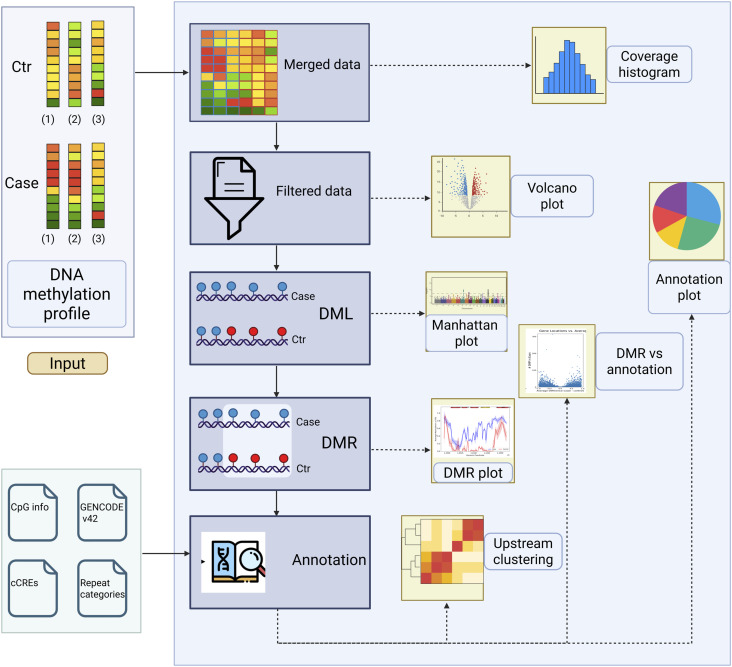
Flowchart of DiffMethylTools. cCRE, candidate cis-regulatory elements; DMR, differentially methylated regions; DML, differentially methylated loci.; (1)–(3): the input methylomes where each row represented a CpG site.

As illustrated in Fig 1, the input of DiffMethylTools is genome-wide methylation profiles of case and control samples with flexible input formats, and then, differentially methylated loci (DMLs) are detected by comparing two groups of methylation profiles. Subsequently, DMLs are clustered into DMRs, followed by functional annotations with genomic features to facilitate biological interpretation. DiffMethylTools also provides a comprehensive set of visualizations for summarizing and interpreting differential DNA methylation patterns.

We evaluated DiffMethylTools and four existing tools using three strategies. We first evaluated their performance on simulation data with known DMLs. We then compared the detection performance of DMLs and DMRs on real data using consensus DMLs and DMRs. After that, we conducted functional annotation analysis, and discussed visualizations offered by our tool.

### Detection performance on simulation data

**Figure 2.**
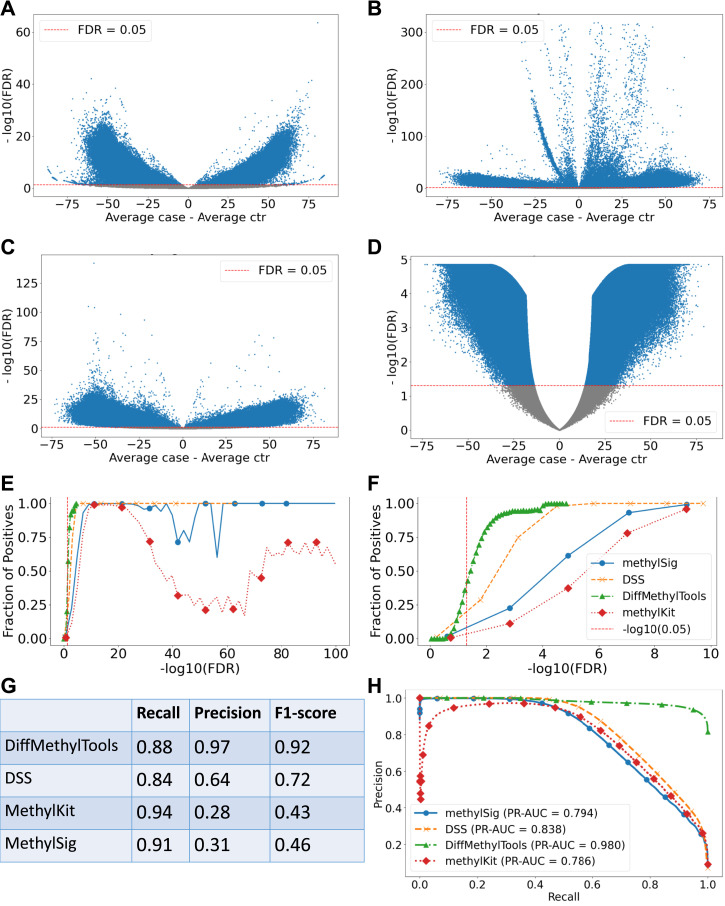
Detection performance of differentially methylated loci (DMLs) by four tools on the simulation data. Volcano plots of detected DMLs by DSS **(A)**, methylKit **(B)**, MethylSig **(C)** and DiffMethylTools **(D)** where x-axis represented the methylation difference of group mean and y-axis represented the negation of logarithm of corrected *P*-values (FDR). Each dot denoted a DML, and a horizontal red line indicated the FDR significance threshold of 0.05. The distribution of known DMLs against FDR was shown in **(E)** for all DMLs and **(F)** for the DMLs with smaller FDR. X-axis represented the negation of logarithm of FDR and y-axis represented the precision of predicted DMLs with FDR smaller than a threshold in x-axis. **(G)** Shows performance of DiffMethylTools alongside DSS, MethylKit and MethylSig. The PR-AUC shown in **(H)** uses −log10(FDR) as ranking score, showing that as the significance (FDR) decreases, how precision (y-axis) and recall (x-axis) changes.

We run all four tools (bsseq was not included because it cannot detect DMLs) on the simulation data with known DMLs. The volcano plots of detected DMLs were illustrated in Fig 2A–D. Different tools had similar range of methylation differences, but their shape of the volcano plots was unique, because they used different statistical models to calculate *P*-values. DiffMethyTools was the most conservative with fewer DMLs with smaller FDR, whereas MethylKit and MethylSig were more dispersed. In particular, MethylKit detected a lot of DMLs whose methylation difference was less than 20, but their FDRs were pretty higher, even higher than a lot of DMLs whose methylation difference was much larger. This indicates potential concerns of detecting too many false positives in MethylKit.

To investigate this situation, we used reliability diagrams by plotting the association of the negation of logarithm of FDRs, i.e., −log10(FDR), against observed frequencies of known DML outcomes. In reliability diagrams, a tool with excellent performance should exhibit a low fraction of known DMLs when — log10(FDR) < −log10(0.05) — reflecting the tool’s ability to avoid false negatives, and large fraction of known DMLs when −log10(FDR) > −log10(0.05) — demonstrating the tool’s effectiveness to accurately identify known DMLs. A perfect model is expected to have a sharp transition around −log10(0.05). In Fig 2E and F, all tools exhibited an increase in the fraction of known DMLs around −log10(0.05). DiffMethylTools and DSS maintained higher percentage of DMLs as −log10(FDR) become larger. The higher percentage of known DMLs by DiffMethylTools first occurred at approximately x = 4, whereas that by DSS was around x = 5, suggesting that DiffMethylTools was more precisely separate significantly and non-significantly methylated loci. In contrast, MethylKit and MethylSig reached their maximums with much smaller FDR, and their percentage of DMLs dropped significantly as the FDR become smaller, suggesting that a lot of detected DMLs by MethylKit and MethylSig were not known DMLs even with significantly smaller FDR.

To quantitatively measure the detection performance, we calculated the recall, precision and F1, as shown in Fig 2G. DiffMethylTools achieved higher F1-score (0.92), which was much higher than DSS (0.72), MethylKit (0.43), and MethylSig (0.46). DiffMethylTools’ precision is 0.97, 0.25 higher than DSS, 0.69 higher than methylKit and 0.66 higher than MethylSig, suggesting that the detected DMLs by DiffMethylTools were reproduced by existing tools, whereas existing tools detected a lot of DMLs which could not be detected by other tools. MethylKit and MethylSig both achieved high recall values (0.94 and 0.91, respectively), but the tradeoff was low precision. Thus, our tool exhibited better performance to detect DMLs. Overall, DiffMethylTools demonstrated higher capabilities to filter out false DMLs whereas maintaining higher recall rates.

We further use PR-AUC with −log10(FDR) as the ranking score (Fig 2H) to evaluate overall performance of precisions and recalls across the full spectrum of significance thresholds. The PR-AUC analysis presented that DiffMethylTools achieved a PR-AUC of 0.98, substantially higher than DSS, methylSig, and methylKit. DiffMethylTools consistently maintained high precision across a wide range of recall values, demonstrating superior performance in prioritizing true DMLs whereas limiting false discoveries. Overall, these results demonstrate that DiffMethylTools yielded a superior performance compared with existing methods.

### Evaluation of DML detection on real methylation data

We then evaluated the detection performance of DMLs on three long-read methylation data and two WGBS data. The F1 scores are summarized in Table 1 for the four tools DiffMethylTools, DSS, MethylKit, and MethylSig, and the detail is described in Tables S1, S2, S3, S4, and S5.

**Table 1.** F1 scores of the detection of differentially methylated loci (DMLs) across five datasets. ONT, Oxford Nanopore sequencing; WGBS, whole-genome bisulfite sequencing.

​	​	DiffMethylTools	DSS	MethylKit	MethylSig
ONT data	HCT116	0.91	0.85	0.66	0.79
	Mφ/Mo	0.48	0.56	0.29	0.33
	AD	0.15	0.05	<0.01	0.04
WGBS	Liver cancer	0.44	0.55	<0.01	0.28
	NK/B cells	0.78	0.88	0.42	0.6

Table S1. Comparison of predicted positions across tools on the dataset of macrophage/monocyte.

Table S2. Comparison of predicted positions across tools on the dataset of HCT116 dataset.

Table S3. Comparison of predicted positions across tools on the dataset of NK/B cells.

Table S4. Comparison of predicted positions across tools on the dataset of liver cancer.

Table S5. Comparison of predicted positions across tools on the AD dataset.

Across all five datasets, DiffMethylTools consistently demonstrated superior performance compared with MethylKit and MethylSig to detect DMLs. On the HCT116 dataset, DiffMethylTools achieved the highest F1 score (0.91), significantly surpassing MethylKit (0.66) and MethylSig (0.79). On the two cell-type datasets, Mφ/Mo and NK/B cells, DiffMethylTools outperformed MethylKit and MethylSig by 0.15–0.19 on Mφ/Mo, and by 0.18–0.36 on NK/B cells. On the two methylation datasets with real human diseases (Alzheimer’s disease and liver cancer), the F1-score of MethylKits and MethylSig was much worse than those achieved by DiffMethylTools. On the liver cancer dataset, DiffMethylTools achieved an F1 score of 0.46, markedly higher than MethylKit (<0.01) and MethylSig (0.28). On the AD dataset, DiffMethylTools achieved a modest F1 score (0.15), outperforming DSS (0.05), MethylSig (0.04), and MethylKit (<0.01). The poor performance of MethylSig was because a lot of detected DMLs by MethylSig could not be reproduced by other tools (Tables S1, S2, S3, S4, and S5). Compared with DSS, DiffMethylTools achieved overall better DML detection on long-read sequencing data and comparable performance across the five datasets. However, as we discussed below, DSS generated much worse performance to detect DMRs than DiffMethylTools.

#### Qualitative evaluation of DMLs

**Figure 3.**
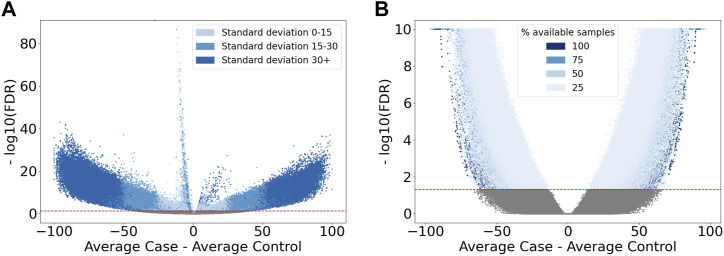
Volcano plots of DMLs detected by methylKit **(A)** and DSS **(B)** on HCT116 data. On (A), DMLs were colored based on their overall SDs (calculated using all samples from both groups), with dark blue indicating high SDs and lighter shades representing lower variability. On (B), DMLs were colored according to how many of the four available samples had enough coverage support for DMLs, with all samples supported (i.e., all four) in dark blue, and less samples in lighter shades.

To investigate the potential issues of DML detection, we visualized DMLs by each tool on the HCT116 data using volcano plots in Fig 3 with methylation difference (Δβ) for x-axis and –log_10_(FDR) as y-axis.

As shown in Fig 3A and B, MethylKit and MethylSig detected a lot of DMLs. MethylKit detected a lot of DMLs with smaller Δβ or smaller overall SD of methylation levels across all samples in the dataset (Fig 3A). Δβ demonstrates methylation difference between two groups and overall SD is calculated using all samples from both groups, suggesting the separation of methylation between two groups. Thus, it is hard to distinguish a DMLs with smaller Δβ or smaller overall SD from random changes, and those DMLs contained more noises. On the other hand, DSS detected a lot of DMLs whose coverage was lower in samples (Fig 3B).

**Figure 4.**
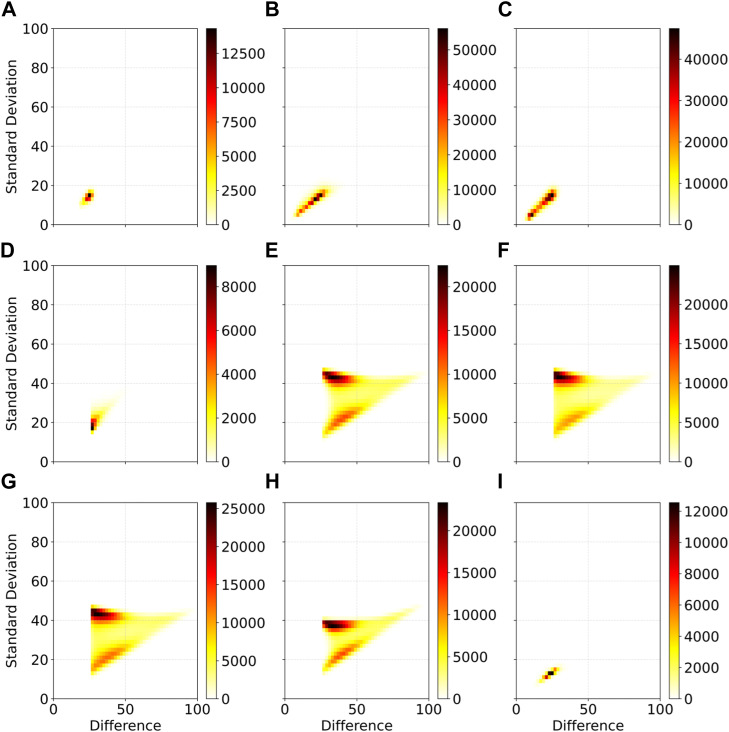
Comparative 2D density histograms of DML detection. The panels visualize the relationship between absolute methylation difference between two groups (x-axis) and the SD (y-axis) calculated using samples from both groups. Color intensity indicates the density of loci within each region, preserving information regarding the relative size of each locus set whereas avoiding overplotting. **(A, B, C)** SOTA-Specific Loci: DMLs identified exclusively by (A) DSS, (B) MethylKit, and (C) MethylSig but rejected by DiffMethylTools. (D) DiffMethylTools unique loci: DMLs uniquely detected by DiffMethylTools and not by any existing SOTA method. **(E, F, G)** Tool Intersections: Shared DMLs between DiffMethylTools and (E) DSS, (F) MethylKit, and (G) MethylSig. **(H)** Consensus: DMLs consistently identified by DiffMethylTools and the intersection of all SOTA tools. **(I)** SOTA consensus: loci identified by the intersection of all three SOTA methods but excluded by DiffMethylTools.

To further characterize the differences between DMLs detected by DiffMethylTools and existing state-of-the-art (SOTA) methods, we visualized the relationship between absolute methylation difference and SD in Fig 4. In Fig 4, we first examined DMLs identified exclusively by each SOTA method but not by DiffMethylTools: Fig 4A–C: DMLs detected by DSS, MethylKit, and MethylSig, respectively, but not detected by DiffMethylTools. Those DMLs visualized within the lower-left region of the plots correspond to both small between-group methylation differences (less than 20) and lower values for SD (less than 30). In contrast, those DMLs uniquely identified by DiffMethylTools (DiffMethylTools − SOTA, Fig 4D) exhibited substantially larger methylation differences, typically exceeding 25%. Although DMLs with larger methylation differences cannot be assumed to represent true biologically functional DMRs, they are generally more likely to reflect robust and biologically relevant group-associated methylation changes than DMLs with smaller differences. Therefore, DiffMethylTools may identify DMLs with greater biological reliability than existing tools.

We next investigated the DMLs shared between DiffMethylTools and each individual SOTA method (DiffMethylTools ∩ DSS in Fig 4E; DiffMethylTools ∩ MethylKit in Fig 4F; DiffMethylTools ∩ MethylSig in Fig 4G), as well as the consensus DMLs identified across all methods (DiffMethylTools ∩ SOTA in Fig 4H). These shared DMLs consistently clustered at larger methylation differences between groups, indicating that agreement across methods occurs primarily for strong signals for differential methylation. In contrast, DMLs identified by SOTA tools but omitted by DiffMethylTools in Fig 4I were tightly clustered in a region characterized by low methylation differences (less than 25%) and low SDs (less than 20), demonstrating that DiffMethylTools effectively filtered out marginal, low-significance methylation sites that were more likely background noise rather than robust biological signals. Together, these results demonstrate that DiffMethylTools preferentially captures reliable differential methylation events whereas excluding those loci detected by existing approaches with weak differential signals.

In summary, different tools used various statistical models and detected DMLs with various characteristics. But our proposed method DiffMethylTools exhibited better capability to capture greater methylation differences and higher SDs in DMLs, and thus achieved overall better performance.

### Investigate DMR detection on real methylation data

#### DMR detection performance

We also evaluated DMR detection for five tools, i.e., DiffMethylTools, DSS, MethylKit, MethylSig, and bsseq, on the five datasets. To benchmark these tools, we found the overlap DMR regions detected by 3+ tools on a dataset as the consensus DMRs (as described in the Methods section). These consensus DMRs on each data were used to calculate precision, recall and F1-score of each tool. In real-world applications, a subset of differential methylation patterns would usually be selected as downstream inputs, and it was very important to get a higher precision set of DMRs. Thus, we investigated the precision of each method in Table 2, and reported recall and F1 scores in Tables S6, S7, S8, S9, and S10.

**Table 2.** Precision of DMR detection by the five tools on the three ONT data and two WGBS data. ONT, Oxford Nanopore sequencing; WGBS, whole-genome bisulfite sequencing. The best performance for each dataset is highlighted in bold.

​	​	DiffMethylTools	DSS	MethylKit	MethylSig	bsseq
ONT data	HCT116	**0.68**	0.61	0.09	0.06	0.64
	Mφ/Mo	**0.84**	<0.01	<0.01	<0.01	0.1
	AD	**0.21**	0.07	<0.01	<0.01	0.01
WGBS	Liver cancer	0.13	<0.01	<0.01	0.02	**0.19**
	NK/B cells	0.71	0.74	0.02	0.04	**0.84**

Table S6. Performance of detecting differentially methylated regions (DMRs) on the AD methylation data using a consensus-based strategy.

Table S7. Performance of detecting differentially methylated regions (DMRs) on the liver-cancer methylation data using a consensus-based strategy.

Table S8. Performance of detecting differentially methylated regions (DMRs) on the macrophage/monocyte methylation data using a consensus-based strategy.

Table S9. Performance of detecting differentially methylated regions (DMRs) on the NK/B cells methylation data using a consensus-based strategy.

Table S10. Performance of detecting differentially methylated regions (DMRs) on the HCT116 methylation data using a consensus-based strategy.

As shown in Table 2, DiffMethylTools consistently outperformed the other methods on long-read data. For example, on HCT116, the precision of DiffMethylTools was 0.68, 0.07 higher than DSS, 0.59 higher than methylKit, 0.62 higher than MethylSig and 0.04 higher than bsseq. On the AD dataset, DiffMethylTools’ precision (0.21) was 0.14 higher than DSS and 0.2 higher than methylKit, MethylSig and bsseq. Similarly, on the Mφ/Mo data, DiffMethylTools’ precision was 0.84, which was 0.74 higher than bsseq and 0.83 higher than DSS, methylKit, and MethylSig. On the two short-read methylation data, DiffMethylTools performance was lower than the best-performed existing tool bsseq, but still ranked as the second or third best positions.

To test the evaluation on the same benchmark, we evaluated Metilene (46) on the same data that are used to test DiffMethylTools. Detailed results are presented in Tables S6, S7, S8, S9, and S10. DiffMethylTools achieved higher F1-scores across all datasets except the liver cancer dataset. Overall, DiffMethylTools outperformed Metilene with an average improvement of 0.24. These results demonstrated that DiffMethylTools achieved overall better detection performance for DMRs and highlighted the robustness and generalizability of DiffMethylTools across different sequencing platforms and biological conditions.

#### Functional annotation analysis of DMRs

##### The distribution of functional annotations

DiffMethylTools offered a comprehensive annotation for DMLs and DMRs by intersecting methylation patterns with various genomic annotations as well as interpretable visual summaries for genomic feature distributions with two types of visualization: a site-based visualization and an annotation-based visualization. A site-based visualization counted the number of CpG sites in methylation patterns that overlapped with an annotation category and thereby reflected the relative concentration or density of methylation signal within different genomic features. In contrast, an annotation-based visualization counted the occurrence of genome annotation that overlapped with methylation patterns. This dual-level resolution revealed notable differences in interpretation.

**Figure 5.**
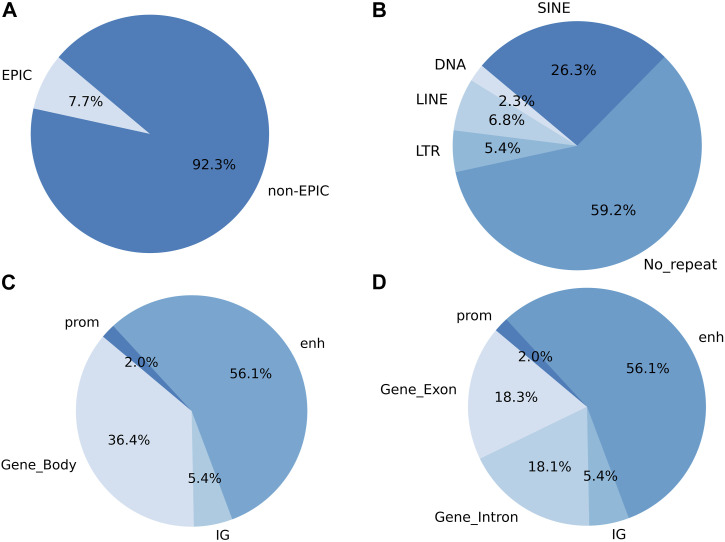
Site-based visualization of DMRs and genomic annotations on NK and B cells. **(A, B, C, D)** Pie charts display the proportional CpG distribution overlapped DMRs in various genomic regions: CpG sites in EPIC versus non-EPIC array (A), repeat elements (B), and functional annotations (promoters, enhancers, gene body, intergenic) in (C) and (D).

**Figure 6.**
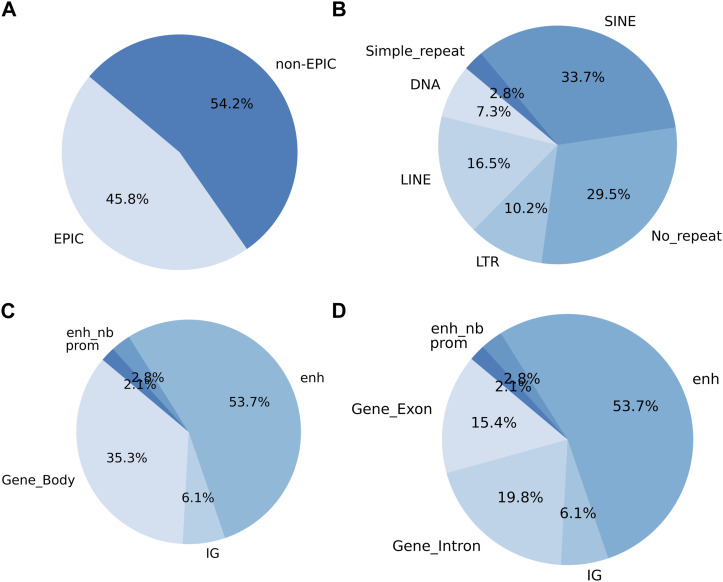
Annotation-based visualization of DMRs between NK and B cells. **(A, B, C, D)** CpG sites in EPIC versus non-EPIC array (A), repeat elements (B), and functional annotations (promoters, enhancers, gene body, intergenic) in (C) and (D).

We illustrated these annotations using the DMRs detected on NK/B cells in Fig 5 (for site-based visualization) and in Fig 6 (annotation-based visualization). We found that 92.3% of sites in DMRs were not detectable in EPIC array (Fig 5A), whereas 54.2% of detected DMRs were completely missed when using EPIC array (Fig 6A). Both suggested that a lot of differential methylation would be missed if EPIC array data were used. Figs 5B and 6B demonstrated that ∼40% CpG sites in DMRs and ∼70% of DMRs were in repeat regions, and these patterns could not be reliably detected if short-read sequencing data were used. In Fig 5C and D as well as Fig 6C and D, ∼53–56% of DMRs were located in enhancers and ∼15–18% in exons, whereas ∼5–6% in intergenic regions. Enhancers and exons were enriched in DMRs compared with that ∼7% of hg38 were defined as cCRE elements whereas 2% for exons. In contrast, intergenic regions (65% of hg38) were depleted. These annotations indicated that the detected DMRs were biologically meaningful. Our tool DiffMethylTools facilitated biological interpretation.

##### Annotation of DMRs

**Figure 7.**
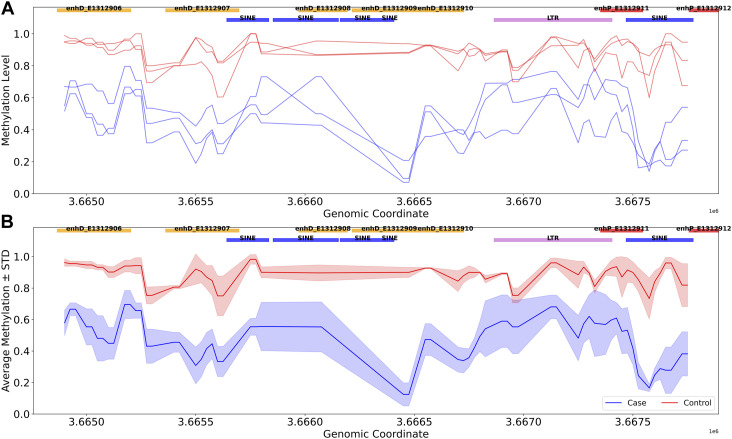
Visualization of an example differentially methylated region (DMR) on chr1:3664875-3667889 identified on NK/B dataset (with NK cells [control] and B cells [case]) by DiffMethylTools. **(A)**: methylation levels for each sample separately; **(B)**: the methylation mean in each group with standard deviation (SD). SINE, short interspersed nuclear elements; LTR, long terminal repeat; EnhP, proximal enhancer with its identifier; enhD, distal enhancer with its identifier.

Besides the visualization of annotation distribution, DiffMethylTools generated high-resolution visualization of each DMR. An example of such visualization was illustrated in Fig 7 for a DMR (chr1:3664875–3667889) detected on the NK/B cells. This visualization not only contained per-sample methylation levels in the DMR (Fig 7A) but also included group-level summary (Fig 7B). Fig 7A revealed a consistent hypermethylation pattern in NK cells compared with B cells, whereas Fig 7B supported this observation with smaller intra-group variability in both conditions, indicating strong reproducibility across replicates. In addition, genomic features and repeat categories that overlapped with the DMR were also illustrated, enabling users to prioritize biologically meaningful DMRs for follow-up analysis and supporting hypothesis-driven exploration of epigenetic regulation. For example, this DMR overlapped an annotated enhancer element (ENSR1_BPMG (47) overlapped with EH38E1312908, EH38E1312909, and EH38E1312910 in Fig 7), which was known to be active in B cells but inactive in NK cells, corroborating the observed methylation differences. These features suggested that this DMR may play a biologically meaningful role in cell-type-specific gene regulation. This integrative visualization clearly demonstrated how DiffMethylTools contextualized epigenetic variation within the broader regulatory and structural genome, facilitating downstream investigation.

### Visualization

**Figure S1.**
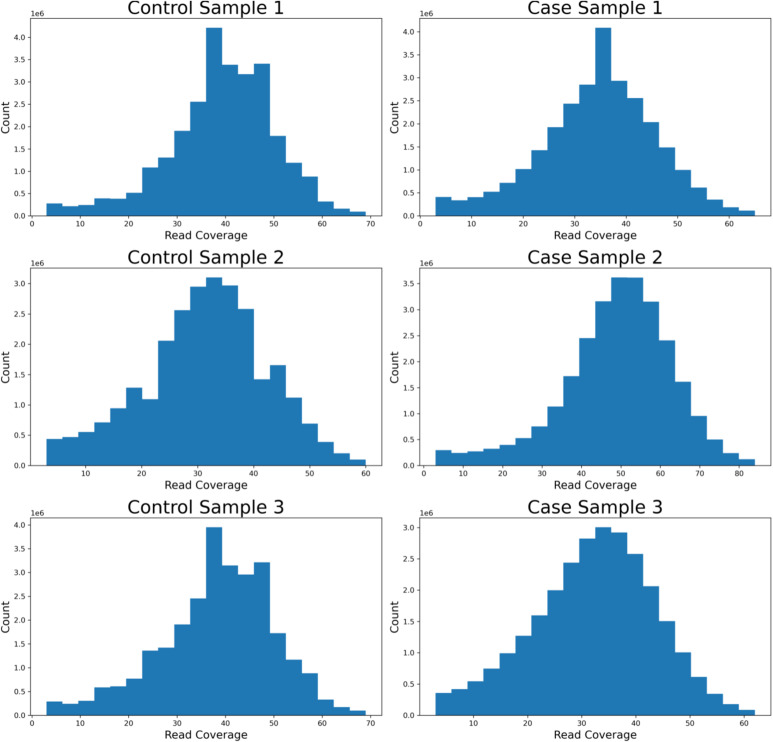
Coverage histogram for NK/B cells sample.

**Figure S2.**
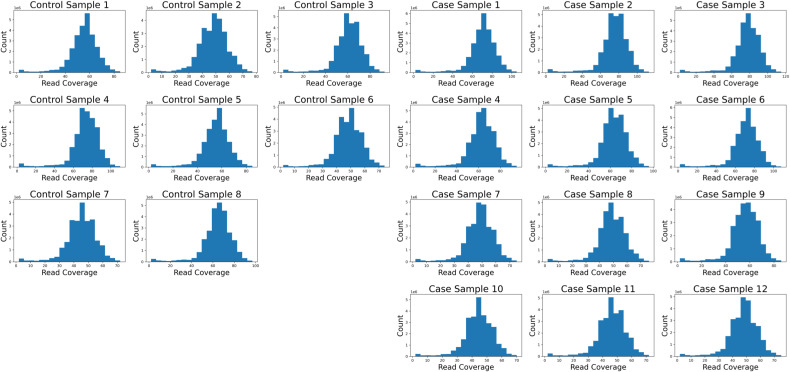
Coverage histogram for AD samples.

**Figure S3.**
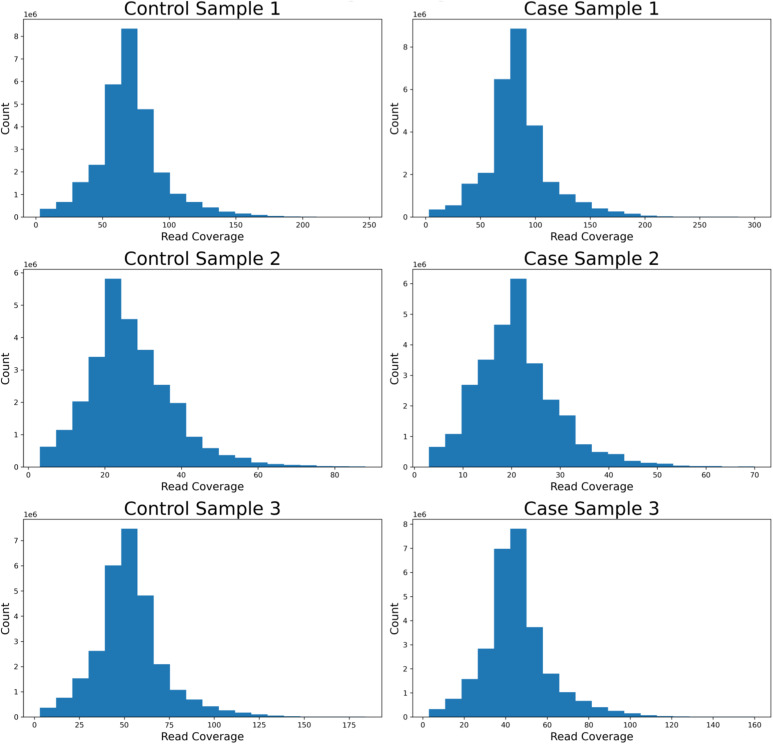
Coverage histogram for macrophage/monocytes cells samples.

**Figure S4.**
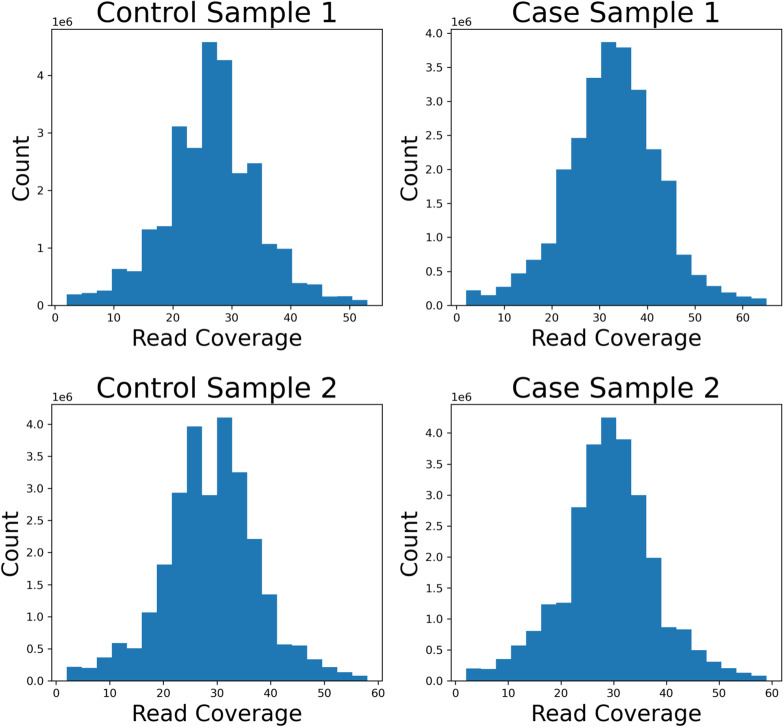
Coverage histogram for HCT116 samples.

**Figure S5.**
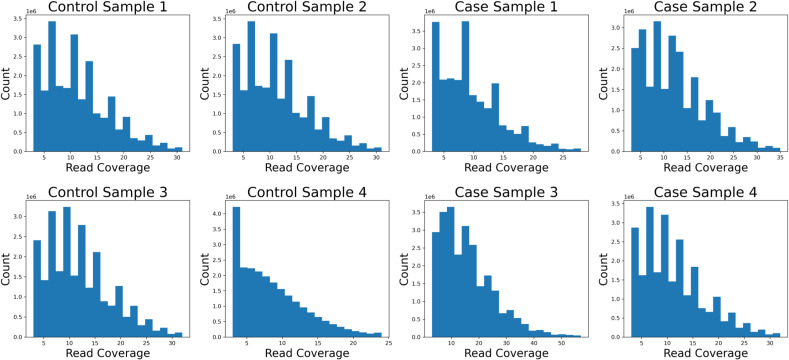
Coverage histogram for liver cancer data.

DiffMethylTools offered diverse visualizations to simplify methylation investigation, as discussed in the Methods section. We discussed several visualizations below and more visualization plots such as per-sample coverage plots could be found in Fig S1, S2, S3, S4, and S5.

**Figure 8.**
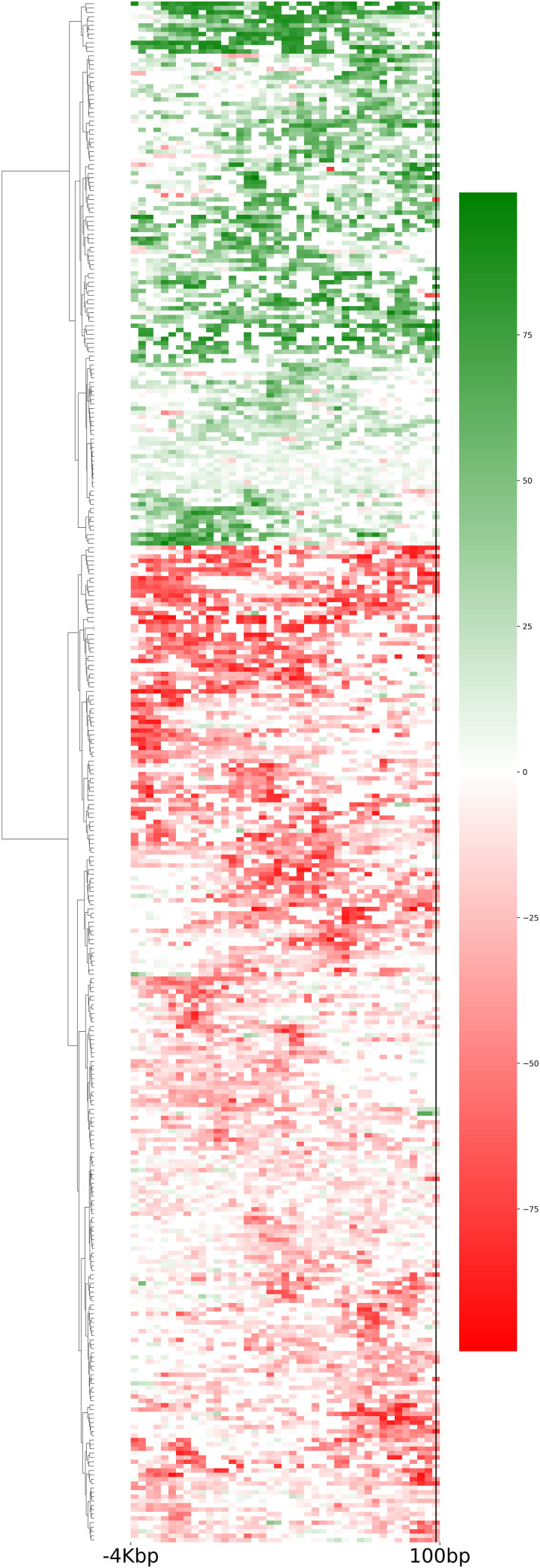
Clustering of upstream regions of genes that overlapped differentially methylated regions (DMRs) in NK/B cell dataset. Each row represented a gene whose upstream contained 1+ DMRs, and each column corresponded to a 100 bp window, and color intensity indicated the methylation difference between two groups after averaging methylation levels for a 100 bp window: red for hypomethylation and green for hypermethylation.

One important visualization was to cluster upstream regions of genes which contained DMRs. This clustering view offered an integrative and interpretable summary of regulatory methylation changes proximally to gene starts and was useful to conduct cross-gene investigation of DMRs. An example was illustrated Fig 8 for the dataset of NK/B cells. As shown in Fig 8, genes’ upstream regions with hypomethylation and hypermethylation were clustered separately. Also, hypomethylation upstream regions were further clustered according to the location of the hypomethylation, suggesting that some genes tend to be hypomethylated in similar relative locations of their upstream regions. This visualization aided in detecting coordinated methylation patterns across gene-proximal regions, exploring how epigenetic shifts might drive transcriptional regulation.

**Figure 9.**
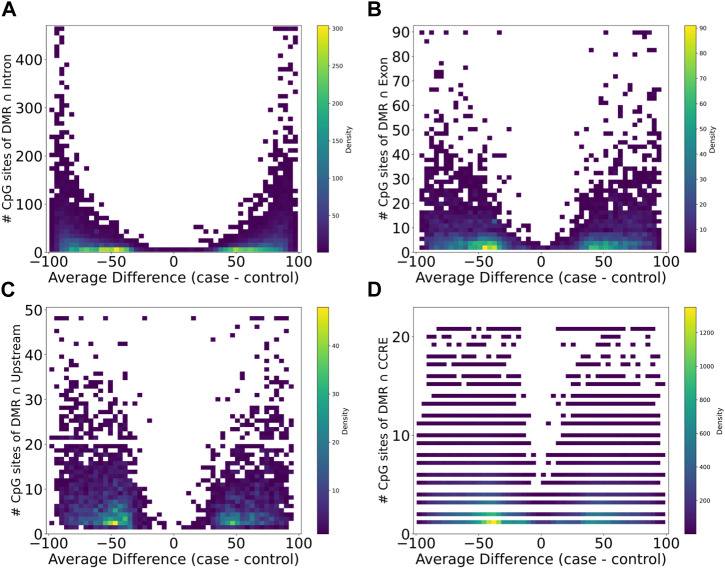
2D density histograms mapping the relationship between average methylation differences and CpG site counts in DMRs. Data are derived from the NK/B cell dataset across four distinct genomic features: **(A)** introns, **(B)** exons, **(C)** upstream regions, and **(D)** candidate cis-regulatory elements (cCREs). The x-axis represents the average methylation difference (case − control), whereas the y-axis denotes the number of CpG sites within the DMR intersecting the respective genomic feature. The color gradient indicates the density of genomic regions within each bin, effectively resolving areas of high spatial overlap.

Besides the clustering of upstream, DiffMethylTools also generated 2D heatmaps of DMRs against introns, exons, upstream regions, and CCRE regions. In Fig 9A–D, x-axis was the average methylation difference between NK cell and B cells, and y-axis was the number of CpG sites in a DMR overlapped with four distinct genomic features. The visualizations presented that the overlapped CpG sites in distinct features varied significantly, and users needed to choose feature-dependent thresholds when investigating DMRs fallen in different genomic features. Furthermore, Fig 9 revealed a distinct bimodal architecture: differentially methylated features strongly polarized toward extreme hypomethylation or hypermethylation, suggesting that these DMRs may have important biological relevance and merit further investigation.

**Figure 10.**
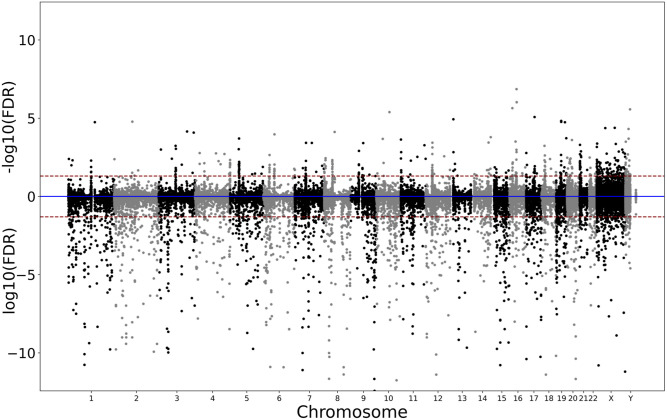
Manhattan plot of DMLs detected on the dataset of the Mφ/Mo dataset. Y-axis: the negative logarithm of FDR (top panel) and the logarithm of FDR (bottom panel); x-axis: the chromosome and genomic coordinates.

Another helpful visualization was the Manhattan plot for the detected DMLs across the genome and one example was presented in Fig 10 for the Mφ/Mo dataset. This plot helped users to quickly visualize the detected DMLs and potential DMLs clusters. Note that this plot might not be used if there were too many DMLs.

## Discussion

Differential methylation biomarkers were critical to investigating epigenetic contributions in various downstream analysis. To facilitate this investigation, we introduced DiffMethylTools, a comprehensive, interpretable, and user-friendly framework that integrates the detection, annotation, and visualization of DMLs and DMRs into a single command. By streamlining this workflow across diverse sequencing technologies, DiffMethylTools enhanced the robust detection of differential methylation compared with existing methods and achieved optimized performance on long-read methylomes. Ultimately, the excellent performance of DiffMethylTools across both synthetic and real data underscored its potential to significantly accelerate epigenomic research and serve as a valuable asset for clinical methylation studies.

Another important feature of DiffMethylTools was its ability to annotate methylation changes with comprehensive genomic context as well as various visualizations. DiffMethylTools mapped DMLs and DMRs to regulatory features, gene elements, and repetitive regions, enabling deeper biological interpretation. This capability was particularly useful to link methylation differences with potential functional effects. Visualizations such as Manhattan plots, gene-centric scatterplots, and per-region methylation heatmaps made DiffMethylTools accessible to both computational and experimental biologists to illustrate and interpret differential methylation.

It is worth noting that whereas our evaluation focused exclusively on 5mC, DiffMethylTools could be used to study other biologically significant epigenetic modifications, such as 5hmC using the same detection process. But users might need to generate their own annotation files for functional annotation and visualization. We have improved the generation of annotation files, and users can easily generate annotations for their specialized investigations.

However, this implementation of DiffMethylTools also has several limitations. First, DiffMethylTools was designed to detect DMRs between two groups. It does not directly support multi-condition (e.g., three or more groups) comparison and pairwise comparison. Future improvements would implement multigroup statistical testing, such as ANOVA or an omnibus F-test, for multi-condition comparisons. Second, DiffMethylTools detected DMLs before clustering them to DMRs. This single site detection may be sensitive to outliers in small sample sizes. It is better to integrate local co-methylation observation (48, 49, 50, 51) to improve the robustness of the DML detection. Third, DiffMethylTools does not consider the confounding effects of genetic variation. Single nucleotide polymorphisms (SNPs) or somatic mutations of CpG sites can lead to zero methylation levels. But this methylation change is mainly because of genetic variants. This effect is more frequently in cancer study, resulting in significant number of false-positive DMLs. Thus, post-processing of removing SNPs from detected DMLs might be needed to eliminate this effect. Finally, the annotation analysis was based on hg38. As major functional annotations are not yet available for the hs1 reference genome, an interim solution would be to use liftover from hg38. In the longer term, annotation for telomere-to-telomere reference genomes needs to be integrated when corresponding resources become available.

## Materials and Methods

### Datasets

#### Whole-genome methylation data

To evaluate the performance of DiffMethylTools and existing tools in detecting differential methylation patterns, we collected five diverse datasets generated by ONT long-read sequencing or bisulfite sequencing short-read sequencing: one ONT data were for a HCT116 cell lines and its knockout of *inositol polyphosphate multikinase* gene (52). This dataset included two biological replicates per condition (HCT116 wide type and knockout). The second ONT dataset was for non-differentiated THP-1 monocytic cells (called Mo) and THP-1 derived macrophage-like cells (Mφ) (53). Each cell type contains three sequencing replicates. The third long-read dataset was for 12 patients with Alzheimer’s disease (AD) and eight unaffected controls. These three datasets were generated by us and the last two were not published. After raw data of Oxford Nanopore techinques were generated using P2-solo and basecalled via ONT’s Dorado basecaller (version 7.2.13+fba8e8925), we used ONT’s modbam2bed to summarize the whole-genome methylation profiling and then computed coverage and methylation percentages using the equations provided below.

$$\text{coverage}=N_{\mathrm{mod}}+N_{\text{canon}}+N_{\text{alt}\;\mathrm{mod}}$$

$$methylation=100*\frac{\;N_{\mathrm{mod}}}{N_{\mathrm{mod}}+N_{\text{alt}\;\mathrm{mod}}+N_{\text{canon}}}$$

where *N*_*mod*_,*N*_canno_ and $N_{\text{alt}\_\mathrm{mod}}$ were count of reads with and without methylation calling as well as with an alternative methylation calling, respectively.

The rest two methylation datasets were generated by short-read bisulfite sequencing technologies. One is the published liver cancer dataset with four liver tumor samples and four-matched control samples (54). And the other was for cell-type samples of the natural killer (NK) versus B-cell, each containing three replicates (55). The sequencing depth information of these datasets is summarized in Figs S1, S2, S3, S4, and S5. Together, these datasets provided a comprehensive benchmark for evaluating the effectiveness and generalizability of detection tools across a range of different sequencing platforms, experimental conditions, and biological contexts.

#### Simulation data

We also generated simulation data with known DMLs to evaluate the performance of differential methylation detection tools. To preserve key biological characteristics observed in real methylomes, we used one methylation data from the HCT116 dataset as a starting methylome to simulate case and control profiles. First, we clustered CpG sites into regions, allowing a maximum gap of 100 base pairs between adjacent sites, and retained only regions containing at least 20 CpG sites. Within each region, methylation levels were smoothed using the LOWESS (locally weighted scatterplot smoothing) (56) algorithm to obtain baseline methylation profiles.

To simulate case methylation data, we introduced diverse methylation changes across the genome. Specifically, we assumed that 40% of CpG sites had minimal change between case and control conditions, 30% had ±5% difference, 10% by ±10% difference, and another 10% by ±15% difference. The remaining sites had larger methylation differences of ±20%, ±30%, ±40%, and ±50%. The simulation methylation percentages for each CpG site were drawn from a truncated normal distribution with the mean equal to the smoothed methylation level ± methylation difference and the SD σ randomly selected from a uniform distribution between 2 and 15, and a random assignment was selected for the direction (±) of methylation change (hyper- or hypo-methylation). We generated three biological replicates for the case group. Three control methylomes were simulated with a similar process except that the mean of the normal distribution is a local smoothed methylation level.

To mimic real methylation data, we also simulated coverages for each CpG site. The simulation coverage for a CpG site was calculated via the equation below using an exponential decay function with Gaussian noise based on the absolute difference between the real and simulated methylation levels.

$$C_{sim}=C_{real}\times e^{\frac{-\left|M_{real}-M_{sim}\right|}{\sigma }}+\epsilon$$

where *C*_*sim*_ was a simulated coverage, *C*_*real*_ was a real coverage from the simulation starting methylome, σ represented the SD of the methylation at a given position, ϵ was a Gaussian noise, *M*_*real*_ was the methylation level from the smoothed reference profile in the control dataset or in the case dataset, and *M*_*sim*_ was the simulated methylation level for that position within the respective datasets. Using this formula, we assumed that the coverage of a CpG site was higher if its methylation level closely matches the average smoothed level. Overall, our simulation strategy yielded methylation and coverage profiles that mimic real sequencing data, providing a robust foundation for benchmarking differential methylation tools.

Note that although benchmarking based on simulation data offer a way to assess the detection performance with a “known” differential methylation in a controlled environment, there are limitations inherent in simulation-based evaluations. First, simulated data are fundamentally constrained by the assumptions and statistical models used during data generation, and thus cannot fully reproduce the complex characteristics of real sequencing data. Second, simulated data may inadvertently favor algorithms whose statistical assumptions align closely with those used during data generation. Thus, the results on simulation data need to be interpreted together with other assessment on real methylation data.

### Flowchart of DiffMethylTools

DiffMethylTools was comprised of several sequential steps, including methylation preprocessing with noise filtering, DML detection, DMR detection, differential methylation annotation as well as differential methylation visualization, as described below.

#### Methylation preprocessing

The inputs of our DiffMethylTools were two groups of methylation profiles in BED format: one group for case samples and the other group for control samples. DiffMethylTools allowed users to provide different formats to define methylation levels and their coverage. This flexibility was useful because preprocessing tools, like Bismark and modbam2bed, usually did not use the same output format, which needed annoying but necessary data preprocessing before differential methylation detection.

The sites from each input methylome were first filtered out if their coverage is less than *c*1. Then, the input methylomes were combined to a matrix $M=\left\{m_{j}^{i}\right\}$ where each row represented a site ***j***, and a column represented a methylome sample ***i*** (**0**≤***i***<***N***), and $m_{j}^{i}$, ranging from 0 to 100, was the methylation percentage (i.e., the ratio of methylation calling over the total coverage for site ***j*** in a sample ***i***), and ***N*** was the number of input samples of two groups. After that, we used another coverage threshold *c*2≥*c*1 to filter out those sites which had less than *t* methylomes with the coverage ≥*c*2 from either the case or control group. The two groups could have different thresholds of *t* if the numbers of methylomes in the two groups were different.

To focus on DML candidates and reduce running time, we filtered out those sites if their SDs of all samples in M were smaller than δ (default 10, adjustable). SDs were calculated using methylome samples from both groups. After that, the missing values were filled with the group means.

#### DML detection

We fed M to a linear regression model implemented in Limma (57). In Limma, empirical Bayesian (eBayes) was used to fit the methylation using groups and other co-factors such as ages if available. A statistical *P*-value was calculated for each site to determine a significant association of methylation differences and the groups. These *P*-values were corrected using multiple testing strategy such as the Benjamini-Hochberg (58) procedure to control the false discovery rate (FDR). A site with corrected *P*-value ≤0.05 was considered as DMLs.

#### DMR detection

Many CpG sites remain proximal in DNA sequences and existing studies found the co-methylation (48, 49, 50, 51) of adjacent CpG sites, suggesting co-localization effect. We thus clustered DMLs into DMRs using the criteria below: (1) a 1,000 bp region contained more than a minimum number of DMLs (default: 3) and their direction of methylation changes was consistent (either all hypomethylated or all hypermethylated), (2) <30% methylation sites within the region exhibited random methylation changes between two groups (the absolute difference of group means <7.5 by default), (3) <10% methylation sites in the region had opposite methylation changes (≥7.5 methylation levels) compared with the DMLs in this region. That is, if the DMLs in the region were hypomethylated, hypermethylation change was the opposite change; otherwise, hypomethylation change was the opposite change. Any regions that did not meet these criteria were split into smaller regions for similar clustering processing. Resultant regions were called DMRs. The clustering strategy was similar to existing tools (26, 30). The thresholds were empirical to control noises effect on DMRs but not optimized. Users can choose their own thresholds for customized detection.

#### Methylation annotations

To facilitate the investigation of biological impact of differential methylation, the detected DMLs and DMRs were annotated with known biological genomic regions using gene annotations from GENCODE (release 42), candidate cis-Regulatory Elements (cCREs) from ENCODE data (59), repeat categories provided by UCSC genome browser (60), and CpG sites from Illumina’s Infinium MethylationChip (HM450, EPIC v1 and v2). These annotations were downloaded and anchored to all 29,401,795 CpG sites in hg38. Then, these annotations were linked to DMLs and DMRs as described below.

The link of DMLs/DMRs to MethylationChip enabled easy validation of differential methylation in epigenome-wide association studies (EWAS) if available. Pie charts were used to show how many DMLs/DMRs could be detectable in EWAS, given that EWAS usually detected <3% of all CpG sites. Annotations of repeats to DMLs/DMRs were useful to investigate potential methylation changes in repeat regions.

Gene annotations of DMLs and DMRs included two components. First, we overlapped different methylation patterns with gene definitions, such as UTR (untranslated region), CDS, introns, exons, upstream regions and intergenic regions. If a methylation pattern overlapped with non-intergenic regions, this pattern was linked with this gene. A pattern could overlap with multiple genes. Second, we overlapped methylation patterns with enhancers and promoters. A gene was annotated with the methylation pattern if it was in 2 kbp adjacency of the promoter or in 50 kbp of the proximal enhancer or in 500 kbp of the distal enhancer. These values were adjustable.

Note that a DMR might be annotated with enhancers or promoters as well as exons, intronic and intergenic regions of multiple genes. An order of precedence [Promoter/Enhancer/Exons > Intronic > Intergenic] was used to exclude low-priority annotations. These annotations were useful because differential methylation had different effects when it occurred in various regions, and identifying the locations with differential methylation provided crucial insights for disease biomarkers and potential epigenetic therapies.

#### Visualization

To facilitate the analysis of differential methylation, DiffMethylTools offered diverse plots to visualize the methylome, DMLs/DMRs and their annotations as shown in Fig 1. Those plots included histogram of coverage, volcano plots of DMLs, Manhattan plot of differential methylation, annotation pie chart, visualization of single DMRs, clustering visualization of DMRs in upstream regions of genes, and gene region versus methylation difference scatter plots. The detail was described below.

• **Histogram of coverage**: Coverage histogram was essential for assessing data quality and guiding filtering decisions. Reliable detection of differential methylation required sufficient read depth. Low coverage increased noise and uncertainty, whereas unusually higher coverage might reflect artifacts such as alignment errors or repetitive regions. DiffMethylTools offered coverage histograms to visualize each sample for filtering high and low coverage sites.
• **Volcano plots of DMLs**: Volcano plots of methylation difference versus FDRs provided easy visualization of significant DMLs. Users could use them to check potential issues in DML detection and to quickly identify the most biologically relevant and statistically significant changes between two groups.
• **Manhattan plot of differential methylation**: Manhattan plot provided a genome-wide view of DMLs by plotting statistical significance (–log10 FDR) against chromosome loci. This visualization enabled users to quickly and visually identify chromosomal loci with clustered epigenetic changes. Such plots were widely used in genome-wide association study (GWAS) data or for known disease loci.
• **Annotation pie chart**: Various pie charts were generated for visualizing annotation of DMRs (recommended) and DMLs. DiffMethylTools generated plots for three major annotation categories: the distribution of CpG sites in EPIC array, repeat elements, and functional genomic features (e.g., promoters, enhancers, gene bodies, and intergenic regions). These visualizations helped to identify the overall distribution of differential methylation.
• **Visualization of single DMRs**: DiffMethylTools provided high-resolution visualizations of individual DMRs, enabling detailed interpretation of locus-specific methylation changes. Two visualization subplots were generated to illustrate per-sample methylation levels or averaged methylation within each group. For the latter subplot, SD within each group was used to illustrate methylation variability. This visualization was also associated with genomic context, overlaying annotated regulatory features (e.g., enhancers) and repetitive elements (e.g., SINEs, LINEs), offering annotation of single DMRs of interest to further investigate biologically meaningful DMRs.
• **Clustering visualization of DMRs in upstream regions of genes**: This clustering view offered an integrative and interpretable summary of how DMRs located in potential promoter regions affected the activities of genes. It enabled users to pinpoint DMR patterns across genes.
• **Gene region vs. methylation difference scatter plots**: DiffMethylTools also generated visualizations that linked differential methylation with specific gene-associated regions. After identifying DMRs, DiffMethylTools grouped methylation levels in 100 bp window, and linked them to intron, exon, upstream, and CCREs using scatter plots. This visualization helped to localize strong methylation shifts in different annotation categories.

### State-of-the-art methods for differential methylation detection

To demonstrate the advantage of DiffMethylTools, we evaluated five widely used existing tools on the same set of datasets for differential methylation detection. These tools included *MethylKit*, *DSS*, *Bsseq*, *MethylSig*, *and Metilene*.

***MethylKit*** was a comprehensive R package specifically designed to analyze DNA methylation data particularly derived from high-throughput bisulfite sequencing technologies. To detect differential methylation for CpG sites or positions, methylKit used Fisher’s exact test for small sample size datasets or low-coverage datasets, or logistic regression to account for multiple covariates or continuous variables. To detect DMRs, MethylKit employed fixed-size windows to segment genome and then identified whether the windows were differentially methylated. Fixed-size windows were not a good solution to detect DMRs because the size of a DMR varies between tens of bp and thousands of bp. MethylKit offered visualization like histograms of coverage and methylation for individual samples, correlation between samples, and hierarchical clustering and principal component analysis (PCA) to cluster samples. But MethylKit heavily relied on the toolkit genomation (1) to annotate DMRs with genomic features such as promoters, exons, introns, and intergenic regions and other functional genomic regions.

***DSS*** *(Differential DNA Methylation Analysis for Sequencing)* was another statistical method to identify DMLs and DMRs. It used a Wald test for beta-binomial distributions to compute *P*-values for DMLs, and then clustered DMLs to DMRs. But DSS does not provide any annotation and visualization functions for detected DMLs and DMRs.

***Bsseq (Bisulfite sequencing analysis)*** used t-statistics to detect DMRs. It incorporated the BSmooth algorithm to smooth methylation estimates across CpG sites, reduce technical noise and increase the signal-to-noise ratio. It defined a DMR region by specifying a minimum number of CpG sites in a genomic region. This region-based method is useful for low-coverage data, where raw methylation estimates may be more variable.

***MethylSig*** was another specialized R package to detect DMLs and DMRs. It employed a beta-binomial regression framework that accounted for biological replication and overdispersion, enabling more accurate identification of DMLs with modest sample sizes. It detected DMRs by clustering significant DMLs based on genomic proximity, or by fixed windows. MethylSig provided scale-aware visualization for CpG-level data and genomic context in narrow regions, and a simplified overview of features and significance in broader regions.

***Metilene*** applies circular binary segmentation to recursively divide the genome into regions of consistent methylation change, and then assesses their statistical significance using a two-dimensional Kolmogorov–Smirnov test. This region-based approach is widely adopted because of its simplicity and minimal preprocessing requirements.

### Evaluation

There were no benchmark DMLs and DMRs in real methylation datasets. We thus provided two ways to evaluate the detection performance of differential methylation. One was based on known DMLs on simulation data. On the simulation data, a position with a Cohen distance of >0.5 was considered differentially methylated. The Cohen distance was calculated using the equation below:

$$d=\frac{{\bar{X}}_{1}-{\bar{X}}_{2}\;}{\sqrt{\frac{\max\left({std}_{1}^{2},7.5\right)+\max\left({std}_{2}^{2},7.5\right)}{2}}}$$

where ${\bar{X}}_{1}$ and ${\bar{X}}_{2}$ were mean methylation of group 1 and group 2, respectively, and std_1_ and std_2_ were the SD of group 1 and group 2, respectively. A minimum variance threshold (of 7.5) was used to mitigate the impact of unrealistically small variance estimates.

Based on known DMLs on simulation data, we used recall, precision and F-1 measures to evaluate the detection performance of each tool.

$$Precision=\frac{TP}{TP+FP}$$

$$Recall=\frac{TP}{TP+FN}$$

$$F1=2\times \frac{Precision\times Recall}{Precision+Recall}$$

where TP was the number of known DMLs which were identified as DMLs by a tool, FP was the number of identified DMLs which were not known DMLs, and FN was the number of known DMLs which were not identified as DMLs by a tool.

On real methylation data, we assumed that a DML was more reliable if it was detected by 2+ tools and considered them as consensus DMLs. We then used the consensus DMLs to calculate precision, recall and F1 for performance comparison. Note that we evaluated the performance of five tools, and to avoid bias, a test tool was not used to generate consensus DMLs.

Similarly, to generate consensus DMRs for real methylation datasets, we employed a consensus-based strategy. Specifically, when we tested the performance of a tool T1 on a dataset, we identified DMRs that were detected by 3+ of the other four tools as consensus DMRs. To account for minor discrepancies, nearby consensus DMRs were merged if they were separated by less than 100 base pairs. Consensus DMRs were trimmed to ensure that both start and end coordinates corresponded to annotated CpG sites. After that, we compared the consensus DMRs against detected DMRs to calculate precision, recall and F1 for performance evaluation, where the overlap of consensus DMRs and detected DMRs was considered as TP, the undetectable regions in consensus DMRs as FN, and the non-consensus regions in detected DMRs as FP. Note that this evaluation strategy was also used by existing tools like Idiffomix (61 *Preprint*), DMRseq (62), Metilene (46), and DMAP (35).

## Supplementary Material

Reviewer comments

## Data Availability

The codes are available on GitHub via https://github.com/qgenlab/DiffMethylTools. ONT methylation data from HCT116 WT and *inositol polyphosphate multikinase* knockout cells are available in the NCBI Sequence Read Archive under BioProject accession PRJNA1255515. Short-read bisulfite sequencing datasets analyzed in this study include a published liver cancer dataset available from the NCBI Gene Expression Omnibus (GEO) under accession GSE70091. DNA methylation data comparing natural killer (NK) cells and B cells are available from GEO under accession GSE186458.

## References

[1] Bird A (2002) DNA methylation patterns and epigenetic memory. Genes Dev 16: 6–21. 10.1101/gad.947102 11782440

[2] Nasrullah, Hussain A, Ahmed S, Rasool M, Shah AJ (2022) DNA methylation across the tree of life, from micro to macro-organism. Bioengineered 13: 1666–1685. 10.1080/21655979.2021.2014387 34986742 PMC8805842

[3] Chowdhury B, Cho IH, Hahn N, Irudayaraj J (2014) Quantification of 5-methylcytosine, 5-hydroxymethylcytosine and 5-carboxylcytosine from the blood of cancer patients by an enzyme-based immunoassay. Anal Chim Acta 852: 212–217. 10.1016/j.aca.2014.09.020 25441900 PMC4254572

[4] Elhamamsy AR (2017) Role of DNA methylation in imprinting disorders: An updated review. J Assist Reprod Genet 34: 549–562. 10.1007/s10815-017-0895-5 28281142 PMC5427654

[5] Sharp AJ, Stathaki E, Migliavacca E, Brahmachary M, Montgomery SB, Dupre Y, Antonarakis SE (2011) DNA methylation profiles of human active and inactive X chromosomes. Genome Res 21: 1592–1600. 10.1101/gr.112680.110 21862626 PMC3202277

[6] Hollister JD, Gaut BS (2009) Epigenetic silencing of transposable elements: A trade-off between reduced transposition and deleterious effects on neighboring gene expression. Genome Res 19: 1419–1428. 10.1101/gr.091678.109 19478138 PMC2720190

[7] Besselink N, Keijer J, Vermeulen C, Boymans S, de Ridder J, van Hoeck A, Cuppen E, Kuijk E (2023) The genome-wide mutational consequences of DNA hypomethylation. Sci Rep 13: 6874. 10.1038/s41598-023-33932-3 37106015 PMC10140063

[8] Booth MJ, Ost TWB, Beraldi D, Bell NM, Branco MR, Reik W, Balasubramanian S (2013) Oxidative bisulfite sequencing of 5-methylcytosine and 5-hydroxymethylcytosine. Nat Protoc 8: 1841–1851. 10.1038/nprot.2013.115 24008380 PMC3919000

[9] Porreca GJ (2010) Genome sequencing on nanoballs. Nat Biotechnol 28: 43–44. 10.1038/nbt0110-43 20062041

[10] Thompson JF, Steinmann KE (2010) Single molecule sequencing with a HeliScope genetic analysis system. Curr Protoc Mol Biol 92: Unit7.10. 10.1002/0471142727.mb0710s92 PMC2954431 20890904

[11] Eid J, Fehr A, Gray J, Luong K, Lyle J, Otto G, Peluso P, Rank D, Baybayan P, Bettman B, (2009). Real-time DNA sequencing from single polymerase molecules. Science 323: 133–138. 10.1126/science.1162986. 19023044

[12] Sereika M, Kirkegaard RH, Karst SM, Michaelsen TY, Sørensen EA, Wollenberg RD, Albertsen M (2022) Oxford nanopore R10.4 long-read sequencing enables the generation of near-finished bacterial genomes from pure cultures and metagenomes without short-read or reference polishing. Nat Methods 19: 823–826. 10.1038/s41592-022-01539-7 35789207 PMC9262707

[13] Lin B, Hui J, Mao H (2021) Nanopore technology and its applications in gene sequencing. Biosensors 11: 214. 10.3390/bios11070214 34208844 PMC8301755

[14] Mazzone R, Zwergel C, Artico M, Taurone S, Ralli M, Greco A, Mai A (2019) The emerging role of epigenetics in human autoimmune disorders. Clin Epigenet 11: 34. 10.1186/s13148-019-0632-2 PMC6390373 30808407

[15] Lakshminarasimhan R, Liang G (2016) The role of DNA methylation in cancer. Adv Exp Med Biol 945: 151–172. 10.1007/978-3-319-43624-1_7 27826838 PMC7409375

[16] Shireby G, Dempster EL, Policicchio S, Smith RG, Pishva E, Chioza B, Davies JP, Burrage J, Lunnon K, Seiler Vellame D, (2022) DNA methylation signatures of Alzheimer’s disease neuropathology in the cortex are primarily driven by variation in non-neuronal cell-types. Nat Commun 13: 5620. 10.1038/s41467-022-33394-7 36153390 PMC9509387

[17] Law PP, Holland ML (2019) DNA methylation at the crossroads of gene and environment interactions. Essays Biochem 63: 717–726. 10.1042/EBC20190031 31782496 PMC6923319

[18] Gao F, Liu X, Wu XP, Wang XL, Gong D, Lu H, Xia Y, Song Y, Wang J, Du J, (2012) Differential DNA methylation in discrete developmental stages of the parasitic nematode Trichinella spiralis. Genome Biol 13: R100. 10.1186/gb-2012-13-10-r100 23075480 PMC4053732

[19] Lv J, Liu H, Su J, Wu X, Liu H, Li B, Xiao X, Wang F, Wu Q, Zhang Y (2012) DiseaseMeth: A human disease methylation database. Nucleic Acids Res 40: D1030–D1035. 10.1093/nar/gkr1169 22135302 PMC3245164

[20] Hu S, Tao J, Peng M, Ye Z, Chen Z, Chen H, Yu H, Wang B, Fan J-B, Ni B (2023) Accurate detection of early-stage lung cancer using a panel of circulating cell-free DNA methylation biomarkers. Biomark Res 11: 45. 10.1186/s40364-023-00486-5 37101220 PMC10134678

[21] Zhang L, Li D, Du F, Huang H, Yuan C, Fu J, Sun S, Tian T, Liu X, Sun H, (2021) A panel of differentially methylated regions enable prognosis prediction for colorectal cancer. Genomics 113: 3285–3293. 10.1016/j.ygeno.2021.07.010 34302946

[22] Yang T, Li C, Wei Q, Pang D, Cheng Y, Huang J, Lin J, Xiao Y, Jiang Q, Wang S, (2024) Genome-wide DNA methylation analysis related to ALS patient progression and survival. J Neurol 271: 2672–2683. 10.1007/s00415-024-12222-6 38372747

[23] Tang J, Xiong Y, Zhou HH, Chen XP (2014) DNA methylation and personalized medicine. J Clin Pharm Ther 39: 621–627. 10.1111/jcpt.12206 25230364

[24] Plant D, Webster A, Nair N, Oliver J, Smith SL, Eyre S, Hyrich KL, Wilson AG, Morgan AW, Isaacs JD, (2016) Differential methylation as a biomarker of response to etanercept in patients with rheumatoid arthritis. Arthritis Rheumatol 68: 1353–1360. 10.1002/art.39590 26814849 PMC4914881

[25] Gaspar JM, Hart RP (2017) DMRfinder: Efficiently identifying differentially methylated regions from MethylC-seq data. BMC Bioinf 18: 528. 10.1186/s12859-017-1909-0 PMC5817627 29187143

[26] Li S, Garrett-Bakelman FE, Akalin A, Zumbo P, Levine R, To BL, Lewis ID, Brown AL, D’Andrea RJ, Melnick A, (2013) An optimized algorithm for detecting and annotating regional differential methylation. BMC Bioinf 14: S10. 10.1186/1471-2105-14-S5-S10 PMC3622633 23735126

[27] Akalin A, Kormaksson M, Li S, Garrett-Bakelman FE, Figueroa ME, Melnick A, Mason CE (2012) methylKit: A comprehensive R package for the analysis of genome-wide DNA methylation profiles. Genome Biol 13: R87. 10.1186/gb-2012-13-10-r87 23034086 PMC3491415

[28] Park Y, Figueroa ME, Rozek LS, Sartor MA (2014) MethylSig: A whole genome DNA methylation analysis pipeline. Bioinformatics 30: 2414–2422. 10.1093/bioinformatics/btu339 24836530 PMC4147891

[29] Wu H, Xu T, Feng H, Chen L, Li B, Yao B, Qin Z, Jin P, Conneely KN (2015) Detection of differentially methylated regions from whole-genome bisulfite sequencing data without replicates. Nucleic Acids Res 43: e141. 10.1093/nar/gkv715 26184873 PMC4666378

[30] Feng H, Conneely KN, Wu H (2014) A Bayesian hierarchical model to detect differentially methylated loci from single nucleotide resolution sequencing data. Nucleic Acids Res 42: e69. 10.1093/nar/gku154 24561809 PMC4005660

[31] Park Y, Wu H (2016) Differential methylation analysis for BS-seq data under general experimental design. Bioinformatics 32: 1446–1453. 10.1093/bioinformatics/btw026 26819470 PMC12157722

[32] Catoni M, Tsang JM, Greco AP, Zabet NR (2018) DMRcaller: A versatile R/Bioconductor package for detection and visualization of differentially methylated regions in CpG and non-CpG contexts. Nucleic Acids Res 46: e114. 10.1093/nar/gky602 29986099 PMC6212837

[33] Sun D, Xi Y, Rodriguez B, Park HJ, Tong P, Meong M, Goodell MA, Li W (2014) MOABS: Model based analysis of bisulfite sequencing data. Genome Biol 15: R38. 10.1186/gb-2014-15-2-r38 24565500 PMC4054608

[34] Dolzhenko E, Smith AD (2014) Using beta-binomial regression for high-precision differential methylation analysis in multifactor whole-genome bisulfite sequencing experiments. BMC Bioinf 15: 215. 10.1186/1471-2105-15-215 PMC4230021 24962134

[35] Stockwell PA, Chatterjee A, Rodger EJ, Morison IM (2014) DMAP: Differential methylation analysis package for RRBS and WGBS data. Bioinformatics 30: 1814–1822. 10.1093/bioinformatics/btu126 24608764

[36] Assenov Y, Müller F, Lutsik P, Walter J, Lengauer T, Bock C (2014) Comprehensive analysis of DNA methylation data with RnBeads. Nat Methods 11: 1138–1140. 10.1038/nmeth.3115 25262207 PMC4216143

[37] Müller F, Scherer M, Assenov Y, Lutsik P, Walter J, Lengauer T, Bock C (2019) RnBeads 2.0: Comprehensive analysis of DNA methylation data. Genome Biol 20: 55. 10.1186/s13059-019-1664-9 30871603 PMC6419383

[38] Warden CD, Lee H, Tompkins JD, Li X, Wang C, Riggs AD, Yu H, Jove R, Yuan YC (2013) COHCAP: An integrative genomic pipeline for single-nucleotide resolution DNA methylation analysis. Nucleic Acids Res 41: e117. 10.1093/nar/gkt242 23598999 PMC3675470

[39] Hansen KD, Langmead B, Irizarry RA (2012) BSmooth: From whole genome bisulfite sequencing reads to differentially methylated regions. Genome Biol 13: R83. 10.1186/gb-2012-13-10-r83 23034175 PMC3491411

[40] Hebestreit K, Dugas M, Klein HU (2013) Detection of significantly differentially methylated regions in targeted bisulfite sequencing data. Bioinformatics 29: 1647–1653. 10.1093/bioinformatics/btt263 23658421

[41] Piao Y, Xu W, Park KH, Ryu KH, Xiang R (2021) Comprehensive evaluation of differential methylation analysis methods for bisulfite sequencing data. Int J Environ Res Public Health 18: 7975. 10.3390/ijerph18157975 34360271 PMC8345583

[42] Rabiner L, Juang B (1986) An introduction to hidden Markov models. IEEE ASSP Mag 3: 4–16. 10.1109/MASSP.1986.1165342

[43] Yu X, Sun S (2016) HMM-DM: Identifying differentially methylated regions using a hidden Markov model. Stat Appl Genet Mol Biol 15: 69–81. 10.1515/sagmb-2015-0077 26887041

[44] Sun S, Yu X (2016) HMM-Fisher: Identifying differential methylation using a hidden Markov model and Fisher’s exact test. Stat Appl Genet Mol Biol 15: 55–67. 10.1515/sagmb-2015-0076 26854292

[45] Saito Y, Tsuji J, Mituyama T (2014) Bisulfighter: Accurate detection of methylated cytosines and differentially methylated regions. Nucleic Acids Res 42: e45. 10.1093/nar/gkt1373 24423865 PMC3973284

[46] Jühling F, Kretzmer H, Bernhart SH, Otto C, Stadler PF, Hoffmann S (2016) metilene: Fast and sensitive calling of differentially methylated regions from bisulfite sequencing data. Genome Res 26: 256–262. 10.1101/gr.196394.115 26631489 PMC4728377

[47] Regulatory feature: ENSR1_BPMG. https://useast.ensembl.org/Homo_sapiens/Regulation/Summary?db=core;fdb=funcgen;r=1:3664875-3667889;rf=ENSR1_BPMG

[48] Guo S, Diep D, Plongthongkum N, Fung HL, Zhang K, Zhang K (2017) Identification of methylation haplotype blocks aids in deconvolution of heterogeneous tissue samples and tumor tissue-of-origin mapping from plasma DNA. Nat Genet 49: 635–642. 10.1038/ng.3805. 28263317 PMC5374016

[49] Affinito O, Palumbo D, Fierro A, Cuomo M, De Riso G, Monticelli A, Miele G, Chiariotti L, Cocozza S (2020) Nucleotide distance influences co-methylation between nearby CpG sites. Genomics 112: 144–150. 10.1016/j.ygeno.2019.05.007 31078719

[50] Li Y, Zhu J, Tian G, Li N, Li Q, Ye M, Zheng H, Yu J, Wu H, Sun J, (2010) The DNA methylome of human peripheral blood mononuclear cells. PLoS Biol 8: e1000533. 10.1371/journal.pbio.1000533 21085693 PMC2976721

[51] Eckhardt F, Lewin J, Cortese R, Rakyan VK, Attwood J, Burger M, Burton J, Cox TV, Davies R, Down TA, (2006) DNA methylation profiling of human chromosomes 6, 20 and 22. Nat Genet 38: 1378–1385. 10.1038/ng1909 17072317 PMC3082778

[52] Sin Z, Kinnear E, Doshi R, Chatterjee S, Derbel H, Guha P, Liu Q (2025) IPMK depletion influences genome-wide DNA methylation. Biochem Biophys Res Commun 766: 151874. 10.1016/j.bbrc.2025.151874 40300331 PMC12685546

[53] Green ID, Pinello N, Song R, Lee Q, Halstead JM, Kwok CT, Wong ACH, Nair SS, Clark SJ, Roediger B, (2020) Macrophage development and activation involve coordinated intron retention in key inflammatory regulators. Nucleic Acids Res 48: 6513–6529. 10.1093/nar/gkaa435 32449925 PMC7337907

[54] Li X, Liu Y, Salz T, Hansen KD, Feinberg A (2016) Whole-genome analysis of the methylome and hydroxymethylome in normal and malignant lung and liver. Genome Res 26: 1730–1741. 10.1101/gr.211854.116 27737935 PMC5131824

[55] Loyfer N, Magenheim J, Peretz A, Cann G, Bredno J, Klochendler A, Fox-Fisher I, Shabi-Porat S, Hecht M, Pelet T, (2023) A DNA methylation atlas of normal human cell types. Nature 613: 355–364. 10.1038/s41586-022-05580-6 36599988 PMC9811898

[56] Cleveland WS (1979) Robust locally weighted regression and smoothing scatterplots. J Am Stat Assoc 74: 829–836. 10.1080/01621459.1979.10481038

[57] Ritchie ME, Phipson B, Wu D, Hu Y, Law CW, Shi W, Smyth GK (2015) Limma powers differential expression analyses for RNA-sequencing and microarray studies. Nucleic Acids Res 43: e47. 10.1093/nar/gkv007 25605792 PMC4402510

[58] Benjamini Y, Hochberg Y (1995) Controlling the false discovery rate: A practical and powerful approach to multiple testing. J R Stat Soc Ser B Stat Methodol 57: 289–300. 10.1111/j.2517-6161.1995.tb02031.x

[59] Abascal F, Acosta R, Addleman NJ, Adrian J, Afzal V, Ai R, Aken B, Akiyama JA, Jammal OA, Amrhein H, (2020) Expanded encyclopaedias of DNA elements in the human and mouse genomes. Nature 583: 699–710. 10.1038/s41586-020-2493-4 32728249 PMC7410828

[60] Perez G, Barber GP, Benet-Pages A, Casper J, Clawson H, Diekhans M, Fischer C, Gonzalez JN, Hinrichs AS, Lee CM, (2025) The UCSC genome browser database: 2025 update. Nucleic Acids Res 53: D1243–D1249. 10.1093/nar/gkae974 39460617 PMC11701590

[61] Majumdar K, Jaffrézic F, Rau A, Gormley IC, Murphy TB (2024) Integrated differential analysis of multi-omics data using a joint mixture model: Idiffomix. arXiv. 10.48550/arXiv.2412.17511 (Preprint posted December 23, 2024).

[62] Korthauer K, Chakraborty S, Benjamini Y, Irizarry RA (2019) Detection and accurate false discovery rate control of differentially methylated regions from whole genome bisulfite sequencing. Biostatistics 20: 367–383. 10.1093/biostatistics/kxy007 29481604 PMC6587918

