# Supplementary material for: DiffMethylTools: a toolbox for the detection, annotation, and visualization of differential methylation
Source: Life Sci Alliance. 2026 Jul 6;9(9):e202603765. doi: 10.26508/lsa.202603765 (PMC13338798; doi:10.26508/lsa.202603765)
Supplement: Supplementary file 4 [file LSA-2026-03765_TableS4.docx]

Table S4. Comparison of predicted positions across tools on the dataset of Liver cancer. For each tool (row), the total number of differential methylation loci (DMLs) with q ≤ 0.05 is shown, along with the number of DMLs identified by at least two other tools, and the size of the intersection between them. Recall, precision, and F1 score were calculated based on overlap with DMLs identified by ≥2 tools. Note: Values <0.01 were denoted accordingly.

|  | **DMLs (q ≤ 0.05)** | **DMLs Identified by ≥2 Other Tools** | **Shared DMLs (Intersection)** | **Recall** | **Precision** | **F1-measure** |
| --- | --- | --- | --- | --- | --- | --- |
| DiffMethylTools | 298264 | 162914 | 102899 | 0.63 | 0.34 | 0.44 |
| DSS | 203761 | 166527 | 103502 | 0.62 | 0.5 | 0.55 |
| MethylKit | 1137607 | 167747 | 815 | <0.01 | <0.01 | <0.01 |
| MethylSig | 608368 | 113484 | 103508 | 0.91 | 0.17 | 0.28 |
