# Supplementary material for: DiffMethylTools: a toolbox for the detection, annotation, and visualization of differential methylation
Source: Life Sci Alliance. 2026 Jul 6;9(9):e202603765. doi: 10.26508/lsa.202603765 (PMC13338798; doi:10.26508/lsa.202603765)
Supplement: Supplementary file 8 [file LSA-2026-03765_TableS8.docx]

Table S8. Performance of detecting differentially methylated regions (DMRs) on the Macrophage/Monocyte methylation data using a consensus-based strategy. For each tool, true positives (TP), false positives (FP), and false negatives (FN) were determined by comparing the tool’s DMRs to consensus DMRs derived from DMRs identified by at least three of the other four tools. Consensus DMRs were generated by merging nearby regions (<100 bp apart) and trimming to annotated CpG boundaries. Precision, recall, and F1-score were calculated based on the overlap with consensus DMRs.

| **Tool** | **TP** | **FP** | **FN** | **Precision** | **Recall** | **F1-score** |
| --- | --- | --- | --- | --- | --- | --- |
| DiffMethylTools | 19788 | 3677 | 3393603 | 0.84 | <0.01 | 0.01 |
| DSS | 31280 | 1132926102 | 0 | <0.01 | 1 | <0.01 |
| bsseq | 26336 | 180011 | 3426822 | 0.12 | <0.01 | 0.014 |
| methylkit | 61123 | 86077836 | 757 | <0.01 | 0.98 | <0.01 |
| methylSig | 31749 | 5346648 | 5024 | <0.01 | 0.86 | 0.011 |
| metilene | 8859 | 1734821 | 3488365 | <0.01 | <0.01 | <0.01 |
